# An online game-based cognitive bias modification for interpretation (CBM-I) program to reduce fear during the COVID-19 pandemic: resilience as a moderator

**DOI:** 10.1080/21642850.2024.2396140

**Published:** 2024-08-28

**Authors:** Wan-Yu Tsai, Yanlin Zhou, Nancy Xiaonan Yu

**Affiliations:** a Department of Social and Behavioural Sciences, City University of Hong Kong, Hong Kong, People’s Republic of China; b Department of Psychology, University of Warwick, Coventry, UK

**Keywords:** CBM-I, fear reduction, COVID-19, resilience, moderation

## Abstract

**Introduction:**

This study examined the training effects of an online game-based cognitive bias modification for interpretation (CBM-I) program in reducing fear during the COVID-19 pandemic in Hong Kong. In addition to investigating the changes in both proximal (i.e. negative and positive interpretations) and distal outcomes (i.e. fear of COVID-19), we examined whether individuals with higher baseline resilience levels would benefit more from the CBM-I program.

**Methods:**

A total of 68 Hong Kong undergraduate students were randomized into either the CBM-I group or a control group, among which 66 participants completed the pretest, post-test, and follow-up on negative and positive interpretations, fear of COVID-19, and resilience.

**Results:**

Compared to the control group, the CBM-I training group showed a significantly greater decrease in negative interpretations, a significantly greater increase in positive interpretations of COVID-19-related ambiguous scenarios, and a trend toward a greater reduction in fear of COVID-19. The CBM-I training was more effective at reducing fear among those with higher levels of resilience at baseline, whereas the control group showed the opposite effect, albeit to a lesser extent.

**Conclusion:**

This online game-based CBM-I training shows the potential to modify the negative interpretation bias toward fear-inducing scenarios and contributes to the reduction of fear. Baseline screening of resilient individuals may optimize the training effects.

## Introduction

Fear is prevalent during the COVID-19 pandemic (Fitzpatrick et al., 2020; Luo et al., 2021; Quadros et al., 2021). Fear of COVID-19 is comorbid with anxiety, traumatic stress, and distress (Şimşir et al., 2022). People in Hong Kong have experienced a moderate level of fear of COVID-19, and those fearing the virus are more likely to be depressed and unhappier (Sit et al., 2021). Negative interpretation bias, which refers to an individual’s tendency to interpret neutral or ambiguous scenarios negatively, has been viewed as an antecedent of fear (Steinman & Teachman, 2014). To reduce fear symptoms, Cognitive Bias Modification for Interpretations (CBM-I) training has been utilized to modify an individual’s negatively biased interpretations of ambiguous scenarios (Gober et al., 2021). CBM-I training is better than exposure therapy in terms of its cost efficiency and accessibility (Ren et al., 2016). For instance, CBM-I can be conducted online without the presence of therapists (Ji et al., 2021), while traditional exposure therapy often requires professional therapists to guide the patient throughout the treatment process (Riddle-Walker et al., 2016). However, previous studies have reported mixed findings about fear reduction following CBM-I. For instance, CBM-I was found to reduce negative interpretation bias and fear when presented with simulated scenarios while showing little fear-reduction effects on actual stressors (Beadel et al., 2016; Steinman & Teachman, 2014). This implies that brief CBM-I training works better in the lab than in real-life contexts. The mixed results may also be attributed to the lack of subgroup analysis concerning who benefits from the training. The current study aimed to examine the effects of CBM-I training on fear reduction during the COVID-19 pandemic and to identify moderating individual differences.

### Negative interpretation bias and fear

According to Reiss’s Expectancy Model of Fear (1991), fear is characterized by two factors. The first factor is an individual’s anticipation of danger or harm in fear-inducing situations. For example, some individuals may expect to contract COVID-19 if they visit a hospital during the pandemic. The second factor is an individual’s underlying explanations that sustain the fear. For instance, an individual may fear contracting COVID-19 due to the belief that it could be fatal. These two factors drive people to avoid fear-inducing situations, which can, in fact, intensify fearful emotions (Hood et al., 2010). The negative beliefs and the self-serving explanations of the negative beliefs could make an individual interpret the fear-related ambiguous scenarios negatively. As Beck and Clark (1997) suggested, people may selectively focus more on the harmful aspects of a situation while overlooking the positive aspects. The tendency to interpret a neutral or ambiguous situation as negative or harmful is known as negative interpretation bias (Beard, 2011). Individuals with this bias are likely to assign negative or harmful interpretations to neutral or ambiguous situations, potentially resulting in fearful emotions (Miles et al., 2009). Given that a negatively biased interpretive style can be an underlying cognitive cause of fear (Steinman & Teachman, 2014), it may be possible to reduce individuals’ fear of COVID-19 by modifying their negative interpretations of COVID-19-related situations.

### CBM-I training

Cognitive bias modification (CBM) represents a suite of interventions developed to directly change biases in cognitive processes, such as attention, memory, and interpretation (Beard, 2011). Among its various subtypes, the CBM-I, or Cognitive Bias Modification for Interpretation, focuses on modifying individuals’ negatively biased interpretations of ambiguous situations that involve negative expectations and high sensitivities toward potential harm (Beard, 2011). By reducing negative interpretations and enhancing positive or benign interpretations, CBM-I training can effectively reduce fear (e.g. Lichtenthal et al., 2017; Steinman & Teachman, 2014). For instance, CBM-I effectively reduces negative interpretation bias and health concerns among cancer survivors (Lichtenthal et al., 2017). This training also reduces height-related interpretation bias among patients with severe acrophobia, decreasing their fear of heights and behavioral avoidance of height-relevant stressors (Steinman & Teachman, 2014).

There are several variations of CBM-I training, with scenario-based CBM-I being one of the most frequently used (Ji et al., 2021). This form of training typically presents participants with an ambiguous scenario that concludes with a partially complete word (Ji et al., 2021), as in the following example: ‘Your family member has contracted COVID-19. Since he/she is under the care of a doctor, he/she will be fi_e.’ The final word of the scenario is intended to resolve the ambiguity positively. Participants receive feedback based on whether they correctly complete the word in a way that resolves the scenario positively. The feedback is designed to increase the likelihood of participants adopting positive interpretations over negative ones. Following the word completion task, a comprehension statement such as ‘Your family member will be fine since they have received proper treatment’ is presented, requiring participants to assess its veracity. The statement serves to reinforce the association between ambiguous scenarios and benign interpretations.

### Mixed findings in CBM-I training effects

CBM-I has been applied to reduce fears as diverse as contamination (Beadel et al., 2016), heights (Steinman & Teachman, 2014), animals (Teachman & Addison, 2008), and disease recurrence (Lichtenthal et al., 2017). The underlying mechanism involves reducing the negative interpretation bias that is related to fearful scenarios (e.g. Beadel et al., 2016). However, the effectiveness of CBM-I in reducing fear has yielded mixed results; some studies report a significant reduction in fear (Lichtenthal et al., 2017; Steinman & Teachman, 2014), while others do not find such effects (e.g. Teachman & Addison, 2008; Beadel et al., 2016). The transfer of learning model (Perkins & Salomon, 1992) suggests that individuals can apply the interpretation style learned in CBM-I training to real-life settings in both near and far transfer modes (Hirsch et al., 2020; LeMoult et al., 2017; Steinman & Teachman, 2014). Near transfer refers to the application of training effect to stimuli related to the training (i.e. proximal outcome; Nahum-Shani et al., 2018), while far transfer involves applying the general interpretation style acquired during training to different real-life scenarios that might subsequently contribute to improvements in distal outcomes (e.g. fear; LeMoult et al., 2017; Nahum-Shani et al., 2018; Steinman & Teachman, 2014). Theoretically, the near-transfer effect should precede the far-transfer effect; thus, in the case of negative interpretation bias as a proximal outcome and fear as a distal outcome, it would be plausible to observe changes in interpretation bias before reduction in fear. The mixed findings from previous studies highlight the need for a careful re-examination of the effects of CBM-I, which should involve multiple assessments using context-appropriate schedules. During the study period, the fifth wave of the pandemic claimed 6,353 lives locally from December 2021 to March 2022 (Cheung et al., 2022). The Hong Kong government imposed policies like closing high-risk places, quarantine, and social isolation (Ho et al., 2021; Wong et al., 2022). Given how alarming COVID-19 was in Hong Kong between late 2021 and early 2022, a shorter follow-up period might have been preferable to mitigate the potential confounding influence of the pandemic’s severity in the local context.

The mixed findings regarding the effects of CBM-I training may also be attributed to a lack of subgroup analysis to determine who benefits from the training and who does not. However, few studies have examined the moderators that influence the effectiveness of CBM-I on fear reduction. For instance, a meta-analysis showed that CBM-I was more effective in community samples than in clinical populations (Cristea et al., 2015). Yet, little focus has been on psychological characteristics that might amplify or diminish the training effects. The theory of vantage sensitivity focuses on individual differences in responsiveness, positing that certain individuals are more sensitive to supportive environmental factors (such as the CBM-I training in the present study) and benefit more from the interventions, whereas others are less responsive or show little improvement due to their inherent characteristics or psychological resources (Pluess & Belsky, 2013). Resilience, as defined by Connor and Davidson (2003), encompasses personal qualities and resources that enable individuals to ‘bounce back’ or recover from adversities. Resilience has been identified as a protective factor against the fear of COVID-19 (Javier-Aliaga et al., 2022). Those with high levels of resilience report lower levels of negative outcomes, such as stress, depression, and anxiety, which are common when individuals experience fear of COVID-19 (e.g. Vos et al., 2021). Despite the buffering effects of resilience on fear of COVID-19, the moderating role of resilience on the effects of CBM-I training on fear reduction is unknown. Therefore, it is worth examining whether individuals with higher baseline resilience levels might benefit more from CBM-I training to experience a greater reduction in fear following the training.

### Present study

This study examined the training effects of an online game-based CBM-I program on fear reduction during the COVID-19 pandemic.

H1. Participants would report a significant decrease in negative interpretations immediately after receiving the CBM-I training.
H2. Participants would show a significant increase in positive interpretations immediately after the CBM-I training.
H3. Participants would experience a significant reduction in fear of COVID-19 in the follow-up after receiving the CBM-I training.

Additionally, as an explorative research question, we investigated the moderating role of baseline resilience in strengthening the training effects. Specifically, we would like to investigate whether participants with higher levels of baseline resilience would show more fear reduction from the baseline to follow-up after receiving the CBM-I training.

## Methods

### Participants

According to Browne (1995), a sample size of 30 participants per group is feasible to examine the effects in a small pilot study (power = 80%, α = .05). Since we had two groups (CBM-I training versus control), we needed at least 60 participants to examine the treatment effects. In addition, we estimated the drop-out rate to be 10% during the pandemic, so a minimum of 66 participants were required for the present study. As the subgroup analysis based on the resilience level was an exploratory research question, we did not estimate the sample size accordingly. A total of 68 undergraduate students were thus recruited, and they were sourced from a Basic Psychology course at the City University of Hong Kong and via social media platforms like WhatsApp and WeChat. Data were collected between November 2021 and February 2022, which was around the fifth wave of the pandemic outbreak in Hong Kong. After providing informed consent, participants were randomly assigned to the CBM-I training group or control group, with a randomization ratio of 1:1, based on their sequence of enrollment in the present study. Participants voluntarily joined the study without any monetary incentives. Students from the Basic Psychology course received course credits for their participation, per their course requirements. The sample consisted of 76.2% women and 23.8% men, with ages ranging from 18 to 26 years (*M*_age_ = 20.57, *SD *= 1.74). Ethical approval for this study was granted by the ethical review board of the Department of Social and Behavioural Sciences, City University of Hong Kong (Ref no.: SSA4708-202110-26).

### Procedures

The study included three-phase assessments. Some participants completed the assessment in paper-and-pencil form in the university psychology lab and library. Others completed the assessment using their laptops, tablets, or computers and were remotely monitored by the first author via Zoom due to the pandemic. At baseline (T1), participants completed the pre-test assessments of interpretation bias (including negative and positive interpretations) toward COVID-19-related scenarios, fear of COVID-19, and resilience.

Participants were randomly assigned to the CBM-I training or control conditions one to three days later. An anxiety imagery prime before CBM-I training has been found to enhance the training effects (Ji et al., 2021). This study adopted such a procedure to activate the fear of the participants and simulate real-life emotional responses to exposure to COVID-19-related stressors. In this anxiety imagery prime procedure, participants from both groups were asked to imagine a COVID-19-related scenario that made them anxious, worried, or scared (e.g. ‘COVID-19 patients cough in front of you,’ ‘people sneeze without their masks on’) and generate a picture of that scenario as vividly as possible before the training. After the anxiety imagery prime, experimental participants completed a scenario-based CBM-I training task on the *Kahoot!* online learning platform. Immediately following the training (T2), they were invited to complete the post-test assessment to assess their negative interpretation bias, positive interpretation bias, and fear of COVID-19. In the control group, followed by the anxiety imagery prime, participants were invited to watch a 40-minute graphic design documentary on YouTube and complete the same post-test assessment as the experimental group.

Five to seven days after the treatment (T3), participants from both groups were contacted again to complete the follow-up assessment to evaluate their negative and positive interpretation biases and fear of COVID-19.

### CBM-I training task

Referring to the scenario-based CBM-I training of Ji et al. (2021), we designed a series of COVID-19-related scenarios and CBM-I training questions. The scenarios included six aspects: public areas, the online or hybrid mode of school life, physical health concerns, COVID-19 news, social distancing and local policies, and vaccination. During the training, participants first saw an ambiguous COVID-19-related scenario. For instance, ‘On a crowded MTR platform, someone in front of you coughs. Since you are wearing a mask, your risk of getting sick is l_w’ (Ji et al., 2021). Each scenario ended with a positive or benign word with a missing letter. Participants chose from four letter options to complete the word. If they chose the correct letter, they saw ‘correct!’ on their computer. Following the word completion task, they were presented with a comprehension statement such as ‘Your risk of getting sick is low since you are wearing a mask,’ and they chose between ‘yes’ and ‘no’ to this comprehension statement. As Ji et al. (2021) stated, the comprehension statements served two purposes: checking the participants’ attention to the depicted scenarios and reinforcing the newly learned interpretative style of the fear-inducing scenarios. The training comprised two parts totaling 134 questions, lasting approximately 40 minutes. Adapted from the single-session CBM-I training procedure of Capron and Schmidt (2016), participants were required to complete the first part of the task, with 64 questions comprising 32 ambiguous scenarios and 32 comprehension questions. During the five-minute break, participants were instructed to draw a tree. This task-shifting activity was designed according to Capron and Schmidt’s (2016) study, wherein participants were asked to complete simple math calculations. To reduce participants’ cognitive burden, we modified this into a tree-drawing activity. In the second part of the task, participants completed 70 questions, comprising 35 ambiguous scenarios and 35 comprehension questions.

We designed and delivered an online game-based CBM-I training program using *Kahoot!*, which is a game-based learning web platform that provides immediate interactive feedback to users and has been widely used in classrooms (Wang & Tahir, 2020). Details can be found in the *Kahoot!* website: https://kahoot.com/ In the present CBM-I training, *Kahoot!* is used to provide efficient feedback to reinforce participants’ desirable responses and reduce their undesirable responses toward COVID-19-related scenarios. Our integration of *Kahoot!* allowed participants to receive CBM-I training via a game with immediate rewards. Each question's available options were presented in different colored boxes. When participants selected the correct box, they would see ‘correct’ immediately and receive points for their correct answer. When participants chose the wrong answer, they would see ‘incorrect’ on the screen and receive no points.

### Measures

#### Negative and positive interpretations

Modifying the Body Sensations Interpretation Questionnaire (Clark et al., 1997), we designed a COVID-19 CBM-I training questionnaire containing 20 ambiguous scenarios to measure negative and positive interpretations. We constructed six aspects of scenarios specific to the pandemic: public areas, the online or hybrid mode of school life, physical health concerns, COVID-19 news, social distancing and local policies, and vaccination. Some scenarios were designed based on the stressors used in studies conducted by Ji et al. (2021) and on virtual reality exposure therapy (Zhang et al., 2020). Each depicted scenario was followed by a negative and a positive interpretation. For instance, under the scenario ‘Your head feels heavy, and you feel hot,’ a negatively biased interpretation was ‘I am developing a COVID-19 symptom,’ and a statement signifying positive interpretation was ‘I didn’t get enough rest.’ Under each scenario, participants were asked to rate the degree to which they agreed with the negative and positive interpretations on a 6-point Likert scale. The depicted scenarios were similar to the CBM-I training scenarios. The negative interpretation subscale had a Cronbach’s α of .81 (T1), .86 (T2), and .89 (T3), and the positive interpretation subscale had a Cronbach’s α of .74 (T1), .85 (T2), and .88 (T3).

#### Fear of COVID-19

The fear of COVID-19 was measured using the Fear of COVID-19 Scale (Ahorsu et al., 2020). The scale contains seven questions on a 4-point Likert scale. Higher scores indicate a higher level of fear of COVID-19. This measure showed a Cronbach’s α of .78 (T1), .84 (T2), and .87 (T3).

#### Resilience

We evaluated resilience at the baseline using the Connor–Davidson Resilience Scale (Connor & Davidson, 2003; Yu & Zhang, 2007), which contains 25 questions based on a 5-point Likert scale. A higher score indicates a higher level of resilience. This measure had a Cronbach’s α of .90.

### Data analyses

We first compared the between-group differences in the study variables at the baseline using the independent t-test. We then conducted a mixed analysis of variance (ANOVA) to test the immediate treatment effects on negative and positive interpretations (H1 and H2, T1 vs. T2) and fear of COVID-19 in the follow-up (H3, T1 vs. T3), with significant group × time interaction effects (*p* < .05) indicating significant training effect. Subsequently, we conducted a moderation analysis using the PROCESS procedure to test the moderating effect of resilience on the training effect on the reduction of fear of COVID-19 at the follow-up (group × baseline resilience). We conducted statistical analyses using SPSS 29.

## Ethics statement

- Institutional Review Board Statement: The study was conducted in accordance with the Declaration of Helsinki and was approved by an Institutional Review Board/Ethics committee. See details under Methods.
- The study received an exemption from an Institutional Review Board/Ethics committee. See details under Methods.

## Results

### Descriptive statistics and baseline comparisons

Among the 68 participants who were recruited to the study, two participants dropped out before completing the experiment. A total of 66 participants completed the experiment, among which three were excluded from data analysis due to being hospitalized, failing to complete the post-test questionnaire, or failing to follow the post-test procedure. In total, valid data for analyses were obtained from 63 participants: 31 from the CBM-I training group and 32 from the control group. The descriptive statistics and baseline comparisons of each variable for the two groups are shown in Table 1. The CBM-I training and control groups showed no significant between-group difference in any of the measures at baseline (all *p *> .05, Table 1).

**Table 1.** Descriptive statistics at three time points and baseline comparisons between the two groups *(N* = 63).

	Experimental group (n = 31) Mean ± SD n (%)	Control group (n = 32) Mean ± SD n (%)	t	*p*
Age Female Negative interpretations	20.42 ± 1.59 21 (67.7%)	20.72 ± 1.89 27 (84.4%)	.68 .15	.50 .13
Baseline	3.18 ± .63	3.15 ± .54	−.17	.87
Post-test	2.89 ± .75	3.03 ± .54	*/*	*/*
Follow-up	2.97 ± .74	3.17 ± .65	*/*	*/*
Positive interpretations				
Baseline	4.21 ± .54	4.25 ± .50	.35	.73
Post-test	4.53 ± .59	4.40 ± .51	*/*	*/*
Follow-up	4.45 ± .63	4.38 ± .58	*/*	*/*
Fear of COVID-19				
Baseline	1.77 ± .45	1.78 ± .50	.06	.95
Post-test	1.54 ± .47	1.66 ± .56	*/*	*/*
Follow-up	1.47 ± .51	1.66 ± .58	*/*	*/*
Resilience (Baseline)	3.38 ± .50	3.43 ± .58	.36	.72

### Training effects

#### Negative interpretations

In terms of negative interpretations of the scenarios, the main effect of time between T1 and T2 was significant (*F*(1, 61) = 20.86, *p *< .001, *η_p_^2 ^*= .26), and the effect of group × time was marginally significant (*F*(1, 61) = 3.43, *p *= .07, *η_p_^2 ^*= .05). The group × time effect between T1 and T3 was significant (*F*(1, 61) = 4.03, *p *= .049, *η_p_^2 ^*= .06), despite the main effect of time being nonsignificant (*F*(1, 61) = 2.99, *p *= .09, *η_p_^2^* = .05). The results suggest a trend of greater reduction of COVID-19-related negative interpretations in the experimental group after the treatment (H1 is supported) and a significantly greater reduction of negative interpretations in T3. The group × time effect on negative interpretations was marginally significant in T2 but significant in T3, implying a time delay before the reduction of negative interpretations manifested rather than immediately after CBM-I training completion. The mixed ANOVA results are shown in Table 2.

**Table 2.** Mixed ANOVA analysis on the training effects between th*e experimental group (n = 31) and control group (n = 32)*.

	F	p	*η_p_^2^*		
**Negative Interpretations**					
Baseline to post-test					
Group	.16	.69	.00		
Time	20.86	< .001	.26		
Group × time	3.43	.07	.05		
Baseline to follow-up					
Group	.34	.56	.01		
Time	2.99	.09	.05		
Group × time	4.03	.049	.06		
**Positive Interpretations**					
Baseline to post-test					
Group	.14	.71	.00		
Time	33.23	< .001	.36		
Group × time	5.51	.02	.08		
Baseline to follow-up					
Group	.02	.88	.00		
Time	19.16	< .001	.24		
Group × time	2.25	.14	.04		
**Fear of COVID-19**					
Baseline to post-test					
Group	.27	.61	.00		
Time	21.50	< .001	.26		
Group × time	2.11	.15	.03		
Baseline to follow-up					
Group	.61	.44	.01		
Time	21.30	< .001	.26		
Group × time	3.60	.06	.06		

Notes: *η_p_^2^ * = partial eta-squared, defined as small (.01), medium (.06), and large (.12) by Cohen (2013).

#### Positive interpretations

As shown in Table 2, the main effect of time was significant in positive interpretations between T1 and T2 (*F* (1, 60) = 33.23, *p *< .001, *η_p_^2 ^*= .36) and between T1 and T3 (*F* (1, 60) = 19.16, *p *< .001, *η_p_^2 ^*= .24). The effect of group × time was significant between T1 and T2 (*F* (1, 60) = 5.51, *p *= .02, *η_p_^2 ^*= .08) but not between T1 and T3 (*F* (1, 60) = 2.25, *p *= .14, *η_p_^2 ^*= .04). These results suggest that the CBM-I training effectively increased the positive interpretations of COVID-19-related scenarios in the experimental group immediately after the training (H2 is supported), although the training effect could not be sustained until the follow-up.

#### Fear of COVID-19

In terms of the reduction of fear of COVID-19 (Table 2), the main effect of time was significant between T1 and T2 (*F*(1, 61) = 21.50, *p *< .001, *η_p_^2 ^*= .26) and between T1 and T3 (*F*(1, 61) = 21.30, *p *< .001, *η_p_^2 ^*= .26). Although the group × time effect was not significant between T1 and T2 (*F*(1, 61) = 2.11, *p *= .15, *η_p_^2^* = .01), a marginally significant group × time effect existed between T1 and T3 (*F* (1, 61) = 3.60, *p *= .06, *η_p_^2 ^*= .06). The non-significant group × time effect in T2 and the marginally significant effect in T3 might imply a time delay for CBM-I effects on fear reduction. This indicated that the level of fear of COVID-19 showed a trend of greater reduction in the CBM-I training group compared to the control group during the follow-up assessment. Therefore, H3 is supported.

### Moderating effect of baseline resilience

**Figure 1.**
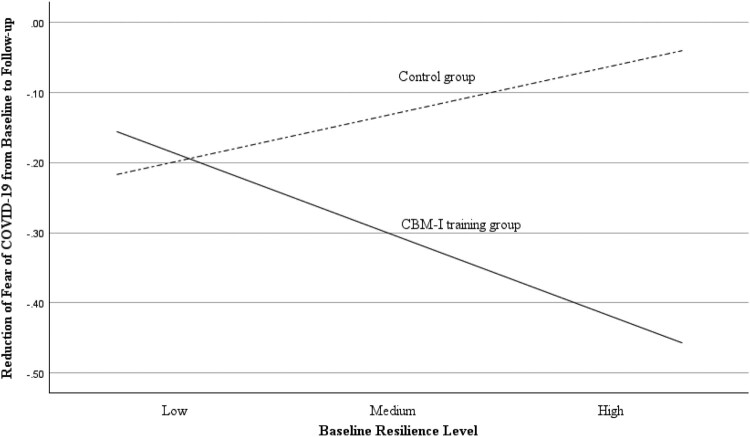
The moderation effect of baseline resilience on the training effect on the reduction of fear of COVID-19 from baseline to the follow-up.

To investigate whether participants with a higher level of baseline resilience benefited more from the CBM-I training, we tested the moderating effect of resilience on the reduction of fear between T1 and T3. Table 3 shows that the baseline resilience significantly moderated the effect of the group on fear reduction (group × baseline resilience: beta (se) = -.44(.17), *p* = .01, 95% CI [−.78, −.11]). The simple slope estimates are shown in Figure 1, indicating the differential effects on fear reduction between the two groups. Specifically, participants in the experimental group with a higher level of baseline resilience showed a greater reduction in fear of COVID-19 from T1 to T3, while the control participants showed a lower level of fear reduction as the levels of resilience increased (medium level: beta (se) = -.18 (.09), *p* = .049, 95% CI [-.25, -.001]; high level: beta (se) = -.42 (.13), *p *= .00, 95% CI [-.67, -.16]). In contrast, the two groups did not show significant differences in fear reduction among those with lower levels of baseline resilience level (beta (se) = .06 (.13), *p* = .63, 95% CI [-.19, .31]). Therefore, our results showed that participants with higher levels of baseline resilience benefited more from the CBM-I training.

**Table 3.** The moderation model of baseline resilience on the training effect on the reduction of fear of COVID-19 from baseline to the follow-up (N = 63).

	*ß*	*SE*	*t*	*p*	95% Confidence Interval	*F*	*R*^2^	*p*
Group	1.33	.58	2.30	.02	[.18, 2.48]	3.64	.16	.02
Baseline resilience	.61	.25	2.41	.02	[.10, 1.11]			
Group × baseline resilience	−.44	.17	−2.64	.01	[−.78, −.11]			
Baseline resilience Low level Medium level High level	.06 −.18 −.42	.13 .09 .13	.49 −2.01 −3.28	.63 .049 .00	[−.19, .31] [−.35, −.00] [−.67, −.16]			

## Discussion

By adopting CBM-I training to modify negative interpretations and further reduce fear of the COVID-19 pandemic, this study benefits from the use of an online game program with a brief single session. Compared to the control group, the CBM-I training group showed a significantly greater decrease in negative interpretations and a significantly greater increase in positive interpretations. Additionally, they showed a trend of a greater reduction in the level of COVID-19-related fear in T3, indicating prolonged benefits rather than an immediate improvement following CBM-I training. This study advanced previous research by examining how individual differences influenced the effectiveness of the training. Notably, resilience was found to moderate the effect of the training on fear reduction; participants with higher levels of baseline resilience experienced a more pronounced decrease in fear. These findings contribute new insights into CBM-I training within the context of the pandemic and underscore the importance of considering individual differences in optimizing training outcomes.

The reduction in the negative interpretations of COVID-19-related ambiguous scenarios and fear of COVID-19 showed similar patterns of presenting desirable changes only until the follow-up assessment, not immediately after the training. Specifically, the CBM-I training group showed a more sustainable reduction in negative interpretations than the control group in the follow-up assessment. On the other hand, the CBM-I training group participants’ fear levels continuously decreased after the intervention to reach a marginally significant level at the follow-up assessment. Our results indicate a possible time delay before the participants can apply their newly learned interpretation styles to fear reduction, which is consistent with the rationale of the transfer of learning that the training effect is reflected in proximal outcomes (i.e. interpretation biases of COVID-19-related scenarios) before contributing to the distal outcome of fear reduction (Perkins & Salomon, 1992). The period between the immediate post-test and the follow-up may allow for the consolidation of new information or skills. For instance, the days following the intervention may expose the participants to different environments or social interactions that reinforce or catalyze the intervention's effects, which only become apparent at the follow-up. In addition, the delayed training effects may be related to the delayed awareness and self-reflection of the participants since sometimes participants may not immediately recognize or be consciously aware of the changes within themselves. The days following the intervention may provide an opportunity for self-reflection, leading to a greater awareness of changes during the follow-up. Future studies are recommended to adopt intensive and longer assessment time points between the post-test and follow-up assessment to capture the temporal and sustainable changes of these proximal and distal outcomes.

Different from these two outcomes, positive interpretations showed an immediate improvement after the training but were not sustained until the follow-up assessment. Although the participants’ increased positive interpretations were not sustained until the follow-up assessment, their fear of COVID-19 continuously decreased until then. Our results indicate that the reduction of fear may be more attributed to the reduction of negative interpretations compared to the enhancement of positive interpretations, which is consistent with previous studies suggesting negative interpretative bias as the root of fear (Steinman & Teachman, 2014). Future CBM-I use is recommended to emphasize reducing negative interpretive styles in the face of potential fear-inducing situations.

Interestingly, participants in the CBM-I training group with a higher level of baseline resilience showed a greater reduction in fear at the follow-up, suggesting that individuals with higher coping resources and readiness to overcome adversities may achieve more desirable outcomes from the CBM-I program. The result provided new insights into the theory of vantage sensitivity by identifying resilience as a moderating factor for the training effect, which explained the variation of the CBM-I training effects across individuals. Because few studies on CBM-I training have reported moderation analysis regarding fear reduction, we call for more investigations to identify other potential moderators (e.g. baseline level of fear, level of catastrophizing, personality traits; de Villiers et al., 2018; Flink et al., 2010) that may strengthen or weaken the training effects. Such endeavors will optimize the training effects by targeting those who benefit more than others.

This study has several limitations. First, the control group watched a documentary, which may have failed to blind the participants from predicting which group they were in. An active control group (e.g. receiving education on the prevention of COVID-19) is desirable to mask the group allocation. Second, the follow-up duration is too short to capture the longer-term training effects of this single-session training. Future studies, particularly those with higher training dosages, may assess the sustainable effects 3 or 6 months later. Fourth, although our results identified resilience as a moderator, the results should be interpreted with caution, given our small sample size. Further research may adopt a larger sample size to investigate the moderating effect of resilience. Last but not least, participants self-reported all the outcomes, and the outcome measures for fear of COVID-19 were simulated scenarios instead of real COVID-10 stressors, given the feasibility. Other objective measures (e.g. skin conductance response; Ren et al., 2016) would be less affected by reporting bias and more reliably reflect the change in fear of COVID-19 among participants.

## Conclusion

The present study demonstrated the near and far transfer effects of an online game-based CBM-I training on fear reduction during COVID-19. In addition, the present study provided insights into the theory of vantage sensitivity by identifying resilience as a moderating factor for the treatment effect. Our findings suggested that the online game-based CBM-I training on COVID-19-related scenarios effectively modified participants’ interpretation bias of ambiguous scenarios, and it is promising for fear reduction. Baseline screening of resilient individuals would optimize the training effects. Future studies may investigate other potential moderators to identify the groups of individuals who benefit most from the CBM-I training.

## Open Scholarship

This article has earned the Center for Open Science badges for Open Data and Open Materials through Open Practices Disclosure. The data and materials are openly accessible at https://drive.google.com/drive/folders/1IU4DyN_GnSw7fTLwPVUn9VbMzMkaiDsR?usp=sharing and https://drive.google.com/drive/folders/1IU4DyN_GnSw7fTLwPVUn9VbMzMkaiDsR?usp=sharing.

## References

[CIT0001] Ahorsu, D. K., Lin, C. Y., Imani, V., Saffari, M., Griffiths, M. D., & Pakpour, A. H. (2020). The fear of COVID-19 scale: Development and initial validation. *International Journal of Mental Health and Addiction*, 1–9. doi:10.1007/s11469-020-00270-8 PMC7100496 32226353

[CIT0002] Beadel, J. R., Ritchey, F. C., & Teachman, B. A. (2016). Role of fear domain match and baseline bias in interpretation training for contamination fear. *Journal of Experimental Psychopathology*, *7*(1), 49–71. doi:10.5127/jep.045414

[CIT0003] Beard, C. (2011). Cognitive bias modification for anxiety: Current evidence and future directions. *Expert Review of Neurotherapeutics*, *11*(2), 299–311. doi:10.1586/ern.10.194 21306216 PMC3092585

[CIT0004] Beck, A. T., & Clark, D. A. (1997). An information processing model of anxiety: Automatic and strategic processes. *Behaviour Research and Therapy*, *35*(1), 49–58. doi:10.1016/S0005-7967(96)00069-1 9009043

[CIT0005] Browne, R. H. (1995). On the use of a pilot sample for sample size determination. *Statistics in Medicine*, *14*(17), 1933–1940. doi:10.1002/sim.4780141709 8532986

[CIT0006] Capron, D. W., & Schmidt, N. B. (2016). Development and randomized trial evaluation of a novel computer-delivered anxiety sensitivity intervention. *Behaviour Research and Therapy*, *81*, 47–55. doi:10.1016/j.brat.2016.04.001 27101256

[CIT0007] Cheung, P. H. H., Chan, C. P., & Jin, D. Y. (2022). Lessons learned from the fifth wave of COVID-19 in Hong Kong in early 2022. *Emerging Microbes & Infections*, *11*(1), 1072–1078. doi:10.1080/22221751.2022.2060137 35348429 PMC9004509

[CIT0008] Clark, D. M., Salkovskis, P. M., Öst, L.-G., Breitholtz, E., Koehler, K. A., Westling, B. E., Jeavons, A., & Gelder, M. (1997). Misinterpretation of body sensations in panic disorder. *Journal of Consulting and Clinical Psychology*, *65*(2), 203–213. doi:10.1037/0022-006X.65.2.203 9086683

[CIT0009] Cohen, J. (2013). *Statistical Power Analysis for the Behavioral Sciences* (Rev. ed.). Academic Press.

[CIT0010] Connor, K. M., & Davidson, J. R. (2003). Development of a new resilience scale: The Connor-Davidson Resilience Scale (CD-RISC). *Depression and Anxiety*, *18*(2), 76–82. doi:10.1002/da.10113 12964174

[CIT0011] Cristea, I. A., Kok, R. N., & Cuijpers, P. (2015). Efficacy of cognitive bias modification interventions in anxiety and depression: Meta-analysis. *British Journal of Psychiatry*, *206*(1), 7–16. doi:10.1192/bjp.bp.114.146761 25561486

[CIT0012] de Villiers, B., Lionetti, F., & Pluess, M. (2018). Vantage sensitivity: A framework for individual differences in response to psychological intervention. *Social Psychiatry and Psychiatric Epidemiology*, *53*(6), 545–554. doi:10.1007/s00127-017-1471-0 29302707 PMC5959990

[CIT0013] Fitzpatrick, K. M., Harris, C., & Drawve, G. (2020). Fear of COVID-19 and the mental health consequences in America. *Psychological Trauma: Theory, Research, Practice, and Policy*, *12*(S1), S17–S21. doi:10.1037/tra0000924 32496100

[CIT0014] Flink, I. K., Boersma, K., & Linton, S. J. (2010). Catastrophizing moderates the effect of exposure in vivo for back pain patients with pain-related fear. *European Journal of Pain*, *14*(8), 887–892. doi:10.1016/j.ejpain.2010.02.003 20219398

[CIT0015] Gober, C. D., Lazarov, A., & Bar-Haim, Y. (2021). From cognitive targets to symptom reduction: Overview of attention and interpretation bias modification research. *Evidence Based Mental Health*, *24*(1), 42–46. doi:10.1136/ebmental-2020-300216 33246935 PMC10231632

[CIT0016] Hirsch, C. R., Krahé, C., Whyte, J., Bridge, L., Loizou, S., Norton, S., & Mathews, A. (2020). Effects of modifying interpretation bias on transdiagnostic repetitive negative thinking. *Journal of Consulting and Clinical Psychology*, *88*(3), 226–239. doi:10.1037/ccp0000455 32068424

[CIT0017] Ho, L. K. K., Fong, C. S., & Wan, T. T. (2021). High level of (passive) compliance in a low-trust society: Hong Kong citizens’ response towards the COVID-19 lockdown. *Policing: A Journal of Policy and Practice*, *15*(2), 1046–1061. doi:10.1093/police/paaa090 PMC7798632 40504216

[CIT0018] Hood, H. K., Antony, M. M., Koerner, N., & Monson, C. M. (2010). Effects of safety behaviors on fear reduction during exposure. *Behaviour Research and Therapy*, *48*(12), 1161–1169. doi:10.1016/j.brat.2010.08.006 20870219

[CIT0019] Javier-Aliaga, D. J., Quispe, G., Quinteros-Zuñiga, D., Adriano-Rengifo, C. E., & White, M. (2022). Hope and resilience related to fear of COVID-19 in young people. *International Journal of Environmental Research and Public Health*, *19*(9), Article 5004. doi:10.3390/ijerph19095004 35564398 PMC9103683

[CIT0020] Ji, J. L., Baee, S., Zhang, D., Calicho-Mamani, C. P., Meyer, M. J., Funk, D., Portnow, S., Barnes, L., & Teachman, B. A. (2021). Multi-session online interpretation bias training for anxiety in a community sample. *Behaviour Research and Therapy*, *142*, Article 103864. doi:10.1016/j.brat.2021.103864 33966880

[CIT0021] LeMoult, J., Colich, N., Joormann, J., Singh, M. K., Eggleston, C., & Gotlib, I. H. (2017). Interpretation bias training in depressed adolescents: Near- and far-transfer effects. *Journal of Abnormal Child Psychology*, *46*(1), 159–167. doi:10.1007/s10802-017-0285-6 PMC5599318 28299526

[CIT0022] Lichtenthal, W. G., Corner, G. W., Slivjak, E. T., Roberts, K. E., Li, Y., Breitbart, W., Lacey, S., Tuman, M., DuHamel, K. N., Blinder, V. S., & Beard, C. (2017). A pilot randomized controlled trial of cognitive bias modification to reduce fear of breast cancer recurrence. *Cancer*, *123*(8), 1424–1433. doi:10.1002/cncr.30478 28055119 PMC5391320

[CIT0023] Luo, F., Ghanei Gheshlagh, R., Dalvand, S., Saedmoucheshi, S., & Li, Q. (2021). Systematic review and meta-analysis of fear of COVID-19. *Frontiers in Psychology*, *12*, Article 661078. doi:10.3389/fpsyg.2021.661078 34177712 PMC8231929

[CIT0024] Miles, A., Voorwinden, S., Mathews, A., Hoppitt, L. C., & Wardle, J. (2009). Cancer fear and the interpretation of ambiguous information related to cancer. *Cognition & Emotion*, *23*(4), 701–713. doi:10.1080/02699930802091116

[CIT0025] Nahum-Shani, I., Smith, S. N., Spring, B. J., Collins, L. M., Witkiewitz, K., Tewari, A., & Murphy, S. A. (2018). Just-in-time adaptive interventions (JITAIs) in mobile health: Key components and design principles for ongoing health behavior support. *Annals of Behavioral Medicine*, *52*(6), 446–462. doi:10.1007/s12160-016-9830-8 27663578 PMC5364076

[CIT0026] Perkins, D. N., & Salomon, G. (1992). Transfer of learning. In T. Husén & T. N. Postlethwaite (Eds.), International encyclopedia of education (2nd ed., pp. 1–13). Oxford: Pergamon Press.

[CIT0027] Pluess, M., & Belsky, J. (2013). Vantage sensitivity: individual differences in response to positive experiences. *Psychological Bulletin*, *139*(4), 901–916. doi:10.1037/a0030196 23025924

[CIT0028] Quadros, S., Garg, S., Ranjan, R., Vijayasarathi, G., & Mamun, M. A. (2021). Fear of COVID 19 infection across different cohorts: A scoping review. *Frontiers in Psychiatry*, *12*, Article 708430. doi:10.3389/fpsyt.2021.708430 34557117 PMC8453018

[CIT0029] Reiss, S. (1991). Expectancy model of fear, anxiety, and panic. *Clinical Psychology Review*, *11*(2), 141–153. doi:10.1016/0272-7358(91)90092-9

[CIT0030] Ren, Z., Lai, L., Yu, X., Li, S., Ruan, Y., & Zhao, L. (2016). Meta-analysis on CBM for anxiety disorder: Effect sizes, moderators and mediation. *Advances in Psychological Science*, *24*(11), 1690–1711. doi:10.3724/SP.J.1042.2016.01690

[CIT0031] Riddle-Walker, L., Veale, D., Chapman, C., Ogle, F., Rosko, D., Najmi, S., Walker, L. M., Maceachern, P., & Hicks, T. (2016). A cognitive behaviour therapy for specific phobia of vomiting (emetophobia): A pilot randomized controlled trial. *Journal of Anxiety Disorders*, *43*, 14–22. doi:10.1016/j.janxdis.2016.07.005 27472452

[CIT0032] Şimşir, Z., Koç, H., Seki, T., & Griffiths, M. D. (2022). The relationship between fear of COVID-19 and mental health problems: A meta-analysis. *Death Studies*, *46*(3), 515–523. doi:10.1080/07481187.2021.1889097 33641626

[CIT0033] Sit, S. M. M., Lam, T. H., Lai, A. Y. K., Wong, B. Y. M., Wang, M. P., & Ho, S. Y. (2021). Fear of COVID-19 and its associations with perceived personal and family benefits and harms in Hong Kong. *Translational Behavioral Medicine*, *11*(3), 793–801. doi:10.1093/tbm/ibab018 33755146 PMC8033593

[CIT0034] Steinman, S. A., & Teachman, B. A. (2014). Reaching new heights: Comparing interpretation bias modification to exposure therapy for extreme height fear. *Journal of Consulting and Clinical Psychology*, *82*(3), 404–417. doi:10.1037/a0036023 24588406 PMC4030404

[CIT0035] Teachman, B. A., & Addison, L. M. (2008). Training non-threatening interpretations in spider fear. *Cognitive Therapy and Research*, *32*(3), 448–459. doi:10.1007/s10608-006-9084-z

[CIT0036] Vos, L. M., Habibović, M., Nyklíček, I., Smeets, T., & Mertens, G. (2021). Optimism, mindfulness, and resilience as potential protective factors for the mental health consequences of fear of the coronavirus. *Psychiatry Research*, *300*, Article 113927. doi:10.1016/j.psychres.2021.113927 33848964 PMC9755114

[CIT0037] Wang, A. I., & Tahir, R. (2020). The effect of using Kahoot! for learning–A literature review. *Computers & Education*, *149*, Article 103818. doi:10.1016/j.compedu.2020.103818

[CIT0038] Wong, S.-C., Au, A. K.-W., Lo, J. Y.-C., Ho, P.-L., Hung, I. F.-N., To, K. K.-W., Yuen, K.-Y., & Cheng, V. C.-C. (2022). Evolution and control of COVID-19 epidemic in Hong Kong. *Viruses*, *14*(11), Article 2519. doi:10.3390/v14112519 36423128 PMC9698160

[CIT0039] Yu, X., & Zhang, J. (2007). Factor analysis and psychometric evaluation of the Connor-Davidson Resilience Scale (CD-RISC) with Chinese people. *Social Behavior and Personality: An International Journal*, *35*(1), 19–30. doi:10.2224/sbp.2007.35.1.19

[CIT0040] Zhang, W., Paudel, D., Shi, R., Liang, J., Liu, J., Zeng, X., Zhou, Y., & Zhang, B. (2020). Virtual reality exposure therapy (VRET) for anxiety due to fear of COVID-19 infection: A case series. *Neuropsychiatric Disease and Treatment*, *16*, 2669–2675. doi:10.2147/NDT.S276203 33192065 PMC7654305

